# Import of Soluble Proteins into Chloroplasts and Potential Regulatory Mechanisms

**DOI:** 10.3389/fpls.2017.00168

**Published:** 2017-02-08

**Authors:** Inga Sjuts, Jürgen Soll, Bettina Bölter

**Affiliations:** ^1^Department Biologie I-Botanik, Ludwig-Maximilians-UniversitätPlanegg-Martinsried, Germany; ^2^Munich Center for Integrated Protein Science CiPSM, Ludwig-Maximilians-UniversitätMunich, Germany

**Keywords:** chloroplast, protein import, TOC, TIC, plastid proteostasis, acclimation

## Abstract

Chloroplasts originated from an endosymbiotic event in which a free-living cyanobacterium was engulfed by an ancestral eukaryotic host. During evolution the majority of the chloroplast genetic information was transferred to the host cell nucleus. As a consequence, proteins formerly encoded by the chloroplast genome are now translated in the cytosol and must be subsequently imported into the chloroplast. This process involves three steps: (i) cytosolic sorting procedures, (ii) binding to the designated receptor-equipped target organelle and (iii) the consecutive translocation process. During import, proteins have to overcome the two barriers of the chloroplast envelope, namely the outer envelope membrane (OEM) and the inner envelope membrane (IEM). In the majority of cases, this is facilitated by two distinct multiprotein complexes, located in the OEM and IEM, respectively, designated TOC and TIC. Plants are constantly exposed to fluctuating environmental conditions such as temperature and light and must therefore regulate protein composition within the chloroplast to ensure optimal functioning of elementary processes such as photosynthesis. In this review we will discuss the recent models of each individual import stage with regard to short-term strategies that plants might use to potentially acclimate to changes in their environmental conditions and preserve the chloroplast protein homeostasis.

## Introduction

Chloroplasts are unique photosynthetic organelles that evolved through an endosymbiotic event ∼1.5 billion years ago. A formerly free-living cyanobacterium was engulfed via phagocytosis by an ancestral eukaryotic host that already contained mitochondria (Gould et al., 2008). During evolution, a dramatic reduction in the bacterial endosymbiont genome size occurred, since 95% of the genes encoding the ∼3000 proteins acting in the chloroplasts were transferred to the host nucleus that attained control over its new organelle. The plastid genome encodes the residual ∼100 genes (Sugiura, 1989; Martin et al., 2002; Timmis et al., 2004). As a consequence, nuclear-encoded chloroplast proteins that were originally encoded on the endosymbiont genome are now translated in the cytosol and are post-translationally translocated into the allocated organelle (Leister, 2003). This translocation process requires a first-sorting event of the so-called preproteins. According to their chloroplast-specific targeting peptide, which is called chloroplast transit peptide (cTP), the preproteins are targeted to the receptor-equipped destination organelle. Previously, this term has been used to define both the (1) N-terminal peptide which is cleaved off in the stroma upon import and (2) the sequence which is necessary and sufficient for import of a cargo protein into the chloroplast. However, these peptides differ from each other, as the sequence of (1) is determined by the processing site and does not contain parts of the mature protein, whereas (2) could also include domains from the mature protein. In order to avoid confusion, Rolland et al tried to find a suitable nomenclature for this issue. The term cTP refers to the sequence of the preprotein which is required for chloroplast targeting and cleaved off upon import. The cTP is determined by the processing site and is not part of the mature protein. In contrast, the sequence which is necessary and sufficient for import of a cargo protein into the chloroplast is called transit peptide. This transit peptide includes the cTP and possibly part of the mature protein (Comai et al., 1988; Bionda et al., 2010; Rolland et al., 2016). During targeting, preproteins can interact with different cytosolic chaperones that enable the cell to keep the preproteins in an unfolded, and hence import-competent, state. After recruiting the chaperoned complexes to the chloroplast outer envelope membrane, translocation is initiated. In the majority of cases, import across the outer and inner envelope membrane of chloroplasts is facilitated by two distinct translocation complexes, called TOC (translocon on the outer chloroplast membrane) and TIC (translocon on the inner chloroplast membrane), respectively. Once inside the stroma, a stromal processing peptidase (SPP) cleaves off the cTP, and the remaining mature protein undergoes folding and insertion or further direction to intraorganeller targets, again with the guidance of stromal chaperones (Richter and Lamppa, 1998; Richter et al., 2005).

It has been long known that import activity correlates with protein demand during plastid development of a plant life (Dahlin and Cline, 1991). In young and fast dividing tissues, the protein demand is especially high, in comparison to adult and non-dividing cell parts. It has been shown that protein import into plastids is developmentally regulated (Li and Teng, 2013). However, since plants are sessile organisms, even mature tissues of a plant are constantly exposed to fluctuating environmental conditions such as temperature and light, and plants must therefore regulate their protein content within the chloroplast to ensure optimal functioning of processes such as photosynthesis. Specifically, the photosynthesis rate depends on different intensities of light and temperature, hence all subunits of the involved complexes have to be produced, imported and assembled according to the current demand.

Several studies noticed that the actual chloroplast proteome is indeed influenced by short-term applications of varying temperature or light conditions, meaning the plant is effectively acclimating upon external stimuli (Dutta et al., 2009; Grimaud et al., 2013). In contrast to adaptation, during which a trait evolves over a longer period of time by means of natural selection, in our understanding acclimation refers to an environmentally inducible and mostly even reversible event which occurs within the organism’s lifetime. Several upstream mechanisms exist, such as changes in the transcription rate of preproteins or involved import receptors upon stress applications. One mentionable example is the upregulation of the TOC GTPase genes upon salt stress in tomato seedlings (Yan et al., 2014). However, these transcriptional mechanisms will not be part of this review; instead, the chloroplast protein import process itself is one advisable target to be highly regulated at different stages, thus leading to a dynamic acclimation of import activity. This acclimation can be achieved by means of post-translational mechanisms such as reversible phosphorylation or oxidation/reduction of both to-be imported and import-related proteins.

Here, we review the individual steps involved in protein translocation into chloroplasts and touch on regulation mechanisms that plants might use to modulate protein import. It is worth mentioning that our understanding of import regulation is still developing. Therefore, we have tried to summarize what is known so far and what the available data from different research groups might mean concerning regulatory mechanisms. These overall speculations might contribute to our current understanding of how plants potentially acclimate to external stimuli by fine-tuning their organellar protein import.

## Cytosolic Sorting of Preproteins and Targeting to the Organelle – the Role of Reversible Phosphorylation

After completion of translation on cytoplasmic ribosomes, the initial step of protein import is the accurate targeting of these newly synthesized preproteins. To avoid mistargeting, chloroplast-destined preproteins harbor an N-terminal cTP that specifically targets them to the chloroplast outer membrane (Bruce, 2001). Unexpectedly, conserved characteristics specific to chloroplast proteins across plant species are missing and the sequences of cTPs are highly heterogeneous in their length and properties. They merely display an overall positive net charge, resulting from the lack of acidic amino acids (Bruce, 2001). Regarding the fact that mitochondrial proteins have specific and conserved features within their N-terminal targeting sequence across plant species, the lack of such a consensus sequence for chloroplast-targeted proteins is striking, thus rendering the question of how specificity for the chloroplast is achieved and mistargeting between these organelles is avoided. One potential hypothesis for the heterogeneity could be different preferences of the preproteins for plastid types, which is determined by distinct cTP features (Li and Teng, 2013).

To sustain import competency by keeping preproteins in an unfolded structure, cytosolic chaperones are involved. Up to now, the most prominent chaperone thought to facilitate appropriate recruiting of preproteins is Hsp70. Both cTPs and the mature part of preproteins have been shown to interact directly with this chaperone, and import activity is clearly stimulated in the presence of Hsp70 (Rial et al., 2000).

**FIGURE 1:**
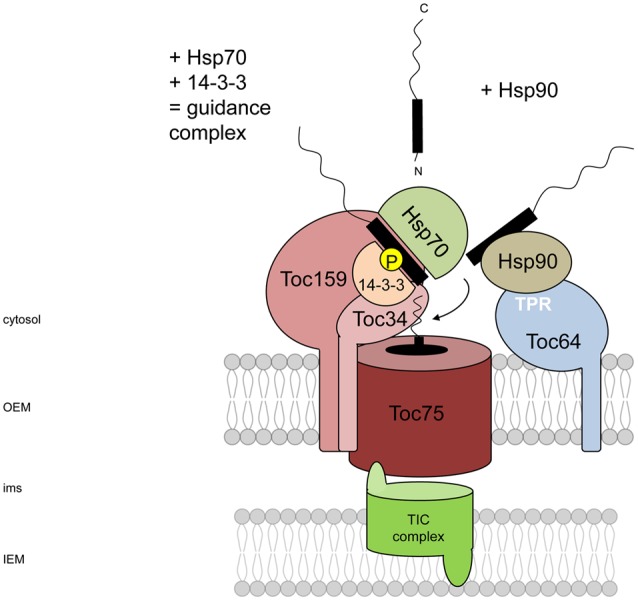
**Chaperone involvement in cytosolic targeting and recognition of preproteins at the outer envelope membrane of chloroplasts.** Preproteins could be chaperoned by the guidance complex or by Hsp90 alone. The guidance complex is represented by Hsp70 that binds to both mature region and cTP of the preprotein and 14-3-3 proteins which bind to the phosphorylated cTP. Hsp70-chaperoned preproteins are recognized by the GTP-dependent receptor proteins Toc159 and Toc34, followed by delivery to the import channel Toc75, whereas precursor proteins bound to Hsp90 are docked to the third receptor Toc64 via its TPR domain and are then handed over to Toc34.

Apart from Hsp70, another component has been identified in cytosolic preprotein targeting: a 14-3-3 protein preferentially binds to phosphorylated serines or threonines within the cTP, which in association with the chaperone Hsp70 leads to increased import efficiency of preproteins. This assembly has been designated the cytosolic guidance complex (May and Soll, 2000) (**Figure 1**). Phosphorylation is mediated by the recently identified STY kinases 7, 18, and 46; a knockout of two and concurrent knockdown of the third kinase led to severe phenotypes in chloroplast biogenesis during greening (Lamberti et al., 2011). However, it seemed that dephosphorylation plays a more crucial role in the actual import process than phosphorylation. It could be shown that under the applied conditions – removal of the phosphorylation site within the binding motif of the cTP for 14-3-3 proteins – the kinetics, rather than the fidelity, of targeting to chloroplasts was impaired (May and Soll, 2000; Nakrieko et al., 2004). In contrast, phosphorylated precursors, or those containing a glutamic acid residue instead to mimic phosphorylation, are only imported very slowly (Waegemann and Soll, 1996). *In vivo* studies showed that an *Arabidopsis* mutant which mimicked the phosphorylated serine in the cTP of the photosynthetic precursor pHcf136 resulted in reduced import activity, and hence impaired photosystem II assembly, most prominent in cotyledons (Nickel et al., 2015). This is probably due to the impossibility of dephosphorylation occurring within the cTP and clearly demonstrates that import and assembly of photosynthetic proteins is highly dependent on a proper phosphorylation/dephosphorylation cycle prior to translocation. Once this process cannot be completed, the chloroplast protein homeostasis is misbalanced.

Like Hsp70, the chaperone Hsp90 is able to bind to both the cTP and mature region of a different subset of preproteins. Its presence alone stimulates protein import into isolated chloroplasts (Qbadou et al., 2006; Fellerer et al., 2011). In contrast to the Hsp70/14-3-3 guidance complex, Hsp90-bound preprotein favors a distinct docking station at the OEM, which will be defined below.

As neither the guidance complex nor the phosphorylation event is essential for successful import, it is highly tempting to speculate that under specific conditions phosphorylation has a regulatory function rather than an essential role in protein import. As phosphorylation is generally a fast response, one can assume that different external stimuli trigger the phosphorylation to regulate protein import. Independent from protein import, this has been shown not only for light-dependent phosphorylation in photosynthetic reactions, but also as a general response to different stress stimuli (Grieco et al., 2016).

It would be interesting to know if the *in vivo* phosphorylation/dephosphorylation circuit of preproteins is enhanced or reduced under stress conditions such as high light treatment, and to define the influence of this regulation mechanism on protein import. Furthermore, whether this effect would be due to either an enhanced activation of the mentioned STY kinases or due to the inhibition of the yet unknown phosphatase remains an interesting question to address.

## Crossing the Outer Envelope Membrane Via the TOC Complex

After synthesis and sorting in the cytosol, the preproteins are recognized at the OEM. This is mainly mediated by the two GTP-dependent receptor proteins Toc34 and Toc159 (Kessler and Schnell, 2009). Both proteins are anchored C-terminally in the OEM and expose their GTP-binding domains toward the cytosol, in consistency with their role as preprotein receptors. Together with a third protein, Toc75, which is deeply embedded in the lipid bilayer and forms the protein conducting channel (Hinnah et al., 1997), they build up a stable complex, resulting in a heterotrimeric TOC core complex (**Figure 1**). Determination of the apparent mass of 500 kDa of the pea multiprotein complex leads to a stoichiometry of 1:4:4 of Toc159/Toc34/Toc75 (Schleiff et al., 2003). Both receptors belong to a plant-specific family of eukaryotic-originated GTPases, sharing some general features. Toc159 is a tripartite protein consisting of three functional domains: an intrinsically disordered acidic domain (A-domain), the GTPase domain (G-domain) and the membrane anchor domain (M-Domain with a mass of ∼54 kDa) (Bölter et al., 1998a; Chen et al., 2000; Richardson et al., 2009). Toc34 contains a cytosolic GTPase domain and is anchored into the OEM by a single transmembrane domain. Both proteins Toc34 and Toc159 bind to distinct regions of the N-terminal cTP, hence they could act simultaneously in receiving preproteins (Becker et al., 2004).

The GTPase activity plays a central role in preprotein recognition and delivery, as non-hydrolyzable GTP analogs inhibit preprotein binding and translocation (Young et al., 1999). Interestingly each individual GTPase domain is dispensable for the plant (Agne et al., 2009; Aronsson et al., 2010), however, a viable plant lacking both domains from both receptors could not yet be isolated. The minimal structure required for sufficient assembly of the TOC complex and to support protein import is the M-domain of Toc159, which can partially complement the loss of Toc159 in *ppi2* mutant plants (Lee et al., 2003).

Toc34 is believed to exist as a homodimer in its GDP-bound state, which exhibits a preprotein-binding site in its GTPase domain (Sun et al., 2002). Upon preprotein delivery, GTPase activity is stimulated and exchanges GDP to GTP. Toc34 in its GTP-bound state binds preproteins with high affinity, which triggers not only the disruption of the Toc34-dimer but also promotes heterodimerization of Toc34 and Toc159. This GTP-heterodimer-complex is now referred to as the active TOC complex (Becker et al., 2004). GTP hydrolysis results in reduced affinity toward the preprotein, the subsequent transfer of the preprotein into the Toc75 channel and the initiation of membrane translocation (Oreb et al., 2007). Taken together, the hypothesized model clearly demonstrates that the receptors are working as GTP/GDP-regulated switches to control preprotein binding and delivery. However, there are still missing factors, such as the GTPase-activating protein or GTP-exchange factor, although it could be shown that peptides from cTPs can stimulate GTPase activity (Jelic et al., 2003).

In *Arabidopsis*, different homologs of the TOC receptors exist, which enhances complexity and specificity toward binding proteins. The Toc159 family consist of four genes, each of them differentially participating in chloroplast biogenesis. These are atToc159, atToc132, atToc120 and atToc90, which show high similarity in their G and M domains, but a high variation in sequence and length of the dynamic A-domain (Bauer et al., 2000; Kubis et al., 2004).

The most abundant isoform is atToc159, consequently the knockout *toc159* (*ppi2*) mutant shows an albino phenotype and is seedling lethal, but can grow hetero-autotrophically (Bauer et al., 2000; Bischof et al., 2011). The latter, and the fact that atToc159 exhibits high expression levels in juvenile developmental stages, led to the suggestion that atToc159 constitutes the primary receptor for photosynthetic precursor proteins, which will be discussed below (Bauer et al., 2000). AtToc90 can complement the albino phenotype of *ppi2* and restores photoautotrophic growth, indicating that atToc90 has a similar function to atToc159 (Infanger et al., 2011). Based on expression pattern and the ability to rescue the *toc159* mutant phenotype, the different TOC receptors are classified in two groups: the above-mentioned group of atToc159 and atToc90, and a second group consisting of atToc132 and atToc120. AtToc132 and atToc120 are expressed at similar levels throughout all tissues and are functionally exchangeable (Ivanova et al., 2004; Kubis et al., 2004). However, atToc120 cannot rescue the phenotype of *toc159*, clearly emphasizing a distinct specificity toward preproteins.

The *Arabidopsis* Toc34 family comprises atToc34 and atToc33, which likewise display differential developmental expression profiles. AtToc33 is highly expressed in juvenile, photosynthetic-active tissues, whereas atToc34 is expressed at low levels throughout all developmental stages and all organs. In line with this expression profile, the *toc33 (ppi1)* mutant showed a pale phenotype during early development, but reached near-WT appearance after 2 weeks of growth. AtToc33 and atToc34 functionally overlap. Different observations led to this conclusion. First, both proteins show 65% sequence similarity; secondly, a small fraction of atToc33 co-immunoprecipitated with atToc120/atToc132, which was originally shown only for atToc34; thirdly, the double knockout of atToc33 and atToc34 is embryo lethal; and last and most critically, atToc34 can complement the *ppi1* phenotype (Jarvis et al., 1998; Ivanova et al., 2004; Kubis et al., 2004).

Different studies led to the overall assumption that various isoforms of the GTPases associate with distinct TOC complexes and may prefer a particular set of precursors. It was suggested that atToc159/atToc90 bind to atToc33, whereas atToc120 and/or atToc132 form a complex together with atToc34 (Ivanova et al., 2004). Originally the idea was favored that the various TOC complexes represent distinct pathways for incoming preproteins. It was stated that the complex consisting of the most abundant isoforms atToc159 and atToc33 preferentially imports highly demanded photosynthetic preproteins, whereas the other TOC isoforms form a translocation complex with specificity toward housekeeping proteins (Ivanova et al., 2004; Kubis et al., 2004; Smith et al., 2004). However, this over-simplified model has been rejected due to a large-scale proteomic and transcriptomic approach by Bischof et al. (2011), in which they identified an import defect for different functional subsets of preproteins in *ppi2* protoplasts. Similar to this observation, equal numbers of photosynthetic and non-photosynthetic preproteins were identified to interact with both atToc159 and atToc132 (Dutta et al., 2014). Nevertheless, the distinct preferential import pathways could be a subtle hint for selectivity of target preproteins, possibly in different developmental stages or under diverse external environmental conditions. As the Toc34 isoforms are functionally interchangeable, preprotein selectivity could be mediated by the Toc159 family. Recent hints are pointing toward a specificity-conferring role of the variable A-domains of the Toc159 receptor family (Inoue et al., 2010).

A third component was identified to assist in receiving preproteins, named Toc64. Its potential role in protein import has been concluded from its ability to bind a precursor protein and the transient association with the other TOC components (Sohrt and Soll, 2000). In contrast to the above-mentioned receptor proteins, Toc64 serves as an initial docking station for Hsp90-bound preproteins und subsequently delivers these preproteins to Toc34 (Qbadou et al., 2006). Toc64 harbors three cytosolic tetratricopeptide repeat (TPR) domains, mediating the interaction with Hsp90 (**Figure 1**). This is a typical feature of proteins interacting with Hsp70/90-associated proteins (Young et al., 2003). The same holds true for a plant ER receptor TPR7 (Schweiger et al., 2012) and interestingly, a Toc64 homolog, namely OM64, was found in plant mitochondria, replacing the mitochondrial TOM70 present in mammals and fungi but absent in plants. Instead, the protein OM64 with a C-terminal TPR domain serves as a receptor for mitochondrial-destined proteins (Chew et al., 2004). Although *in vitro* a strong interaction between Hsp90 and Toc64 could be measured with a *K_D_* of 2.4–15.5 μm (Schweiger et al., 2012) the essentiality of these TPR proteins *in vivo* is still under debate. Since chloroplasts lacking Toc64 sustain their import capacity, it is feasible that this docking protein rather constitutes more an additional regulatory component to the general TOC receptor complex than being an essential constituent. However, it could be shown that atToc33 and Toc64 cooperate in preprotein import, hence it is reasonable to say that atToc33 can overcome the loss of Toc64 function as preproteins are still recognized (Sommer et al., 2013), while only chaperone binding is lost.

After the preprotein has been delivered to the receptor proteins, it has to be translocated through the membrane. The preprotein-conducting channel in the OEM is represented by the beta barrel protein Toc75 (Schnell et al., 1994). The essential nature of Toc75 is demonstrated by its gene being a single copy conserved throughout all plant lineages and the embryo lethality of knockout lines (Jackson-Constan and Keegstra, 2001). The protein belongs to the Omp85 superfamily, which is exclusively found in gram-negative bacteria, mitochondria and plastids (Bölter et al., 1998b). Typically for this family, the structure of Toc75 exhibits two features: 16-18 arranged beta strands forming the C-terminal beta domain, and several POTRA domains at its N-terminus (Clantin et al., 2007). Irrespective of the fact that POTRA domains are required for Toc75 function (Paila et al., 2016), the orientation and thus exact molecular function of these POTRA domains remain a matter of debate. On the one hand, it is assumed that these domains are facing the cytosolic side of the OEM, assisting in preprotein interaction (Sommer et al., 2011). However, a recent study proposed a localization of the POTRA domains in the intermembrane space (Chen et al., 2016).

*In vitro* analyses showed preprotein binding during import and the import process itself being inhibited with Toc75 antibodies (Tranel et al., 1995). Electrophysiological analyses revealed that reconstituted Toc75 in lipid bilayers forms a voltage-gated channel with a pore size of 14Å at its narrowest part (Hinnah et al., 2002). In contrast to the other TOC components, Toc75 harbors an N-terminal bipartite transit peptide. One part directs the protein into the stroma where the SPP cleaves off this portion once the extreme N-terminus reaches the stroma, whereas the second cleavage site is processed by a plastidic type I signal peptidase (Plsp1), which is localized to the IEM (Inoue et al., 2005).

## Crossing the Intermembrane Space and Inner Envelope Membrane Via the TIC Complex

Successful import requires not only the interaction between preproteins and outer membrane receptors, but also the formation of super complexes between the translocons of both OEM and IEM via contact sites that enable the preprotein to pass through both membranes simultaneously (Schnell and Blobel, 1993). Both complexes are facing the intermembrane space, thus some proteins localized in this compartment have to be involved in the import process. However, only limited knowledge about import-related factors of the intermembrane space is available. Presently, the only member identified in this compartment to be involved in protein translocation is the soluble protein Tic22. Tic22 has been shown to interact with preproteins during protein import (Kouranov et al., 1998). Structural and functional studies led to the hypothesis that Tic22 is working as a molecular chaperone, as *Arabidopsis* mutants lacking Tic22 showed growth and biogenesis defect and a decreased import activity (Kasmati et al., 2013; Rudolf et al., 2013). One potential role for Tic22 would be, like the cytosolic counterparts, to ensure proper targeting and prevent misfolding during the transfer between TOC and TIC. However, this role has not been confirmed yet.

The TIC counterpart of the TOC core channel Toc75 is Tic110. Tic110 was the first TIC component described Schnell et al. (1994) and is the second most abundant protein in the IEM (Lübeck et al., 1996). It was found in a supercomplex associated with TOC components and incoming preproteins, suggesting a functional role as the central part of the IEM translocon (Lübeck et al., 1996).

**FIGURE 2:**
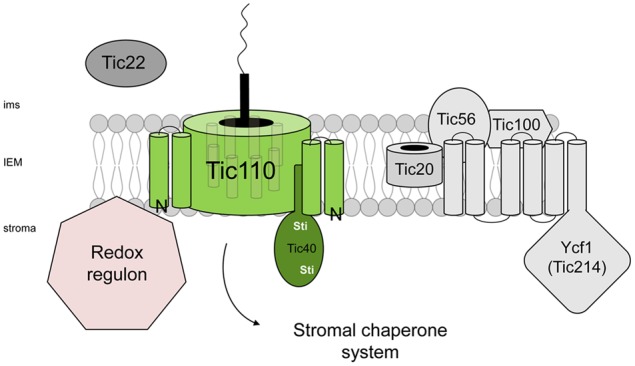
**Crossing the inner envelope membrane of chloroplasts via the TIC complex.** The counterpart of the outer channel protein is the IEM protein Tic110 which is a functional dimer. Two hydrophobic domains anchor the protein into the IEM whereas further eight amphipathic helices are involved in the channel formation. Tic40 is supposed to interact with Tic110 with its Sti1 domain and acts further as a scaffold for stromal chaperones. Controversial, the 1MDa-complex depicted on the right side comprises atTic20 as the channel protein, atTic56 embedded in the complex, atTic100 located at the IMS and the plastid encoded Ycf1 (atTic214) with its six transmembrane domains and a large stromal C-terminus.

Reconstitution of a Tic110-protein lacking its two N-terminal hydrophobic transmembrane stretches (pea sequence: aa91-966, ΔN-110) resulted in a cation-selective channel with a diameter of 1.7 nm, which is similar to the diameter of the channel of Toc75 and hence sufficient for preprotein threading (Heins et al., 2002; Balsera et al., 2009) (**Figure 2**). However, two controversial models concerning the topology and function of Tic110 still persist. Undoubtedly and universally accepted is the fact that the 110-kDa protein is anchored into the membrane by its two N-terminal, highly hydrophobic helices (Inaba et al., 2003; Balsera et al., 2009). In our current topological model, we can combine the essential functions of Tic110, which has been under discussion for a long time. On the one hand, Tic110 assembles into its channel-like structure via its four amphipathic helices, substantiating its function as the main translocation pore. The four membrane-spanning helices consequently lead to the formation of two loops that are extended into the intermembrane space, which could be confirmed by limited proteolysis experiments (Lübeck et al., 1996; Balsera et al., 2009). On the other hand, a large part of the C-terminus is protruding into the stroma and thus could fulfill the additional function of Tic110 acting as a scaffold for chaperones and co-chaperones (Inaba et al., 2005). The crystal structure of a *Cyanidioschyzon merolae* Tic110 version, which consists of the C-terminus including only the last amphipathic helix, is proposed to be too flattened and elongated to form a channel protein (Tsai et al., 2013). However, as it is unlikely that such a shortened protein can fold into its native conformational structure, it is still reasonable to assume that the full-length Tic110 protein is able to build the channel protein via its amphipathic, membrane-spanning helices.

Like Toc75, Tic110 is encoded by a single gene and constitutively expressed in all tissues. Homozygous T-DNA insertion lines are embryolethal, and heterozygous plants already exhibit a clear growth and greening defect, clearly emphasizing the necessity of Tic110 in chloroplast biogenesis and overall plant viability (Kovacheva et al., 2005). Import of Tic110 is achieved by targeting the protein into the stroma and after cleavage of the cTP, Tic110 is re-inserted into the lipid bilayer of the IEM (Vojta et al., 2007).

Using a cross-linking strategy, another TIC component could be directly associated to Tic110, named Tic40. Tic40 consists of a single transmembrane helix which anchors the protein at the IEM, resulting in a large stroma-facing, soluble domain. This C-terminal part harbors two Hip/Hop/Sti domains, building binding sites for Tic110 and the stromal Hsp70/93 chaperones. The main function of Tic40 is to co-chaperone the translocation process of incoming preproteins by coordinating Hsp93 activity (Chou et al., 2006) (**Figure 2**).

A further TIC component, named Tic20, was identified by its ability to covalently cross-link with a precursor protein en route to the chloroplast (Kouranov and Schnell, 1997; Kouranov et al., 1998). Structural prediction indicated three or four hydrophobic transmembrane domains (Kouranov et al., 1998). Tic20 is essential in *Arabidopsis*. Chloroplasts isolated from Tic20 antisense lines are impaired in preprotein import (Chen et al., 2002). In addition, early phylogenetic analysis indicated a relation of Tic20 with bacterial amino acid transporter and cyanobacterial proteins of unknown function suggesting a role as a translocation channel (Reumann and Keegstra, 1999). However, a latter study including many more genomes was unable to reproduce these claims (Gross and Bhattacharya, 2009). Nonetheless, the important role of Tic20 in chloroplast biogenesis is evident and it was proposed early on by Reumann et al. (2005) that Tic20 and Tic110 form independent preprotein translocation channels. Besides this circumstantial evidence for the notion, direct support comes from electrophysiological studies using either heterologously expressed and purified Tic20 (Kovács-Bogdán et al., 2011) or a 1MDa-complex from *Arabidopsis*, of which Tic20 is one constituent (Kikuchi et al., 2009, see below), which both showed the channel-forming capacity of the applied material. Using a cleavable proteinA-tagged variant of Tic20 expressed in transgenic *Arabidopsis* plants, the authors were able to purify the 1MDa complex via affinity purification. The obtained complex contained three other proteins in addition to Tic20: atTic56, atTic100 and atTic214 (Ycf1) (Kikuchi et al., 2013) (**Figure 2**).

Interestingly, Ycf1 is one of the last enigmatic open-reading frames of the chloroplast genome without an assigned function (Drescher et al., 2000). It is predicted to contain at least six transmembrane helices at its N-terminus (de Vries et al., 2015). AtTic56 and atTic100 are nuclear-encoded proteins, the first deeply embedded in the holo-complex without any predicted transmembrane domain, whereas the latter is supposed to associate with the complex on the intermembrane space site (Kikuchi et al., 2013). However, major questions came up concerning the exact physiological roles of the involved proteins. So far, for the potential involvement of Tic100, no data are available. However, for atTic56, a proteomic analysis showed that most of the chloroplast proteins are still imported into the organelle in *atTic56* mutant plants, pointing toward a still functioning import machinery (Köhler et al., 2015). Furthermore, an alternative role independent from protein import for atTic56 was suggested, since Köhler et al. established a link between processing of plastid rRNA and the assembly of plastid ribosomes. They stated that a defect in plastid ribosome construction is responsible for the albino phenotype of *atTic56-1* mutant plants, thus leading to a potential role of atTic56 in ribosome assembly and establishment of a functional plastid translation machinery (Köhler et al., 2016). Even more importantly, since Ycf1 is missing not only in all grasses but also in a variety of dicotyledonous plants, one can speculate about its overall significance in protein import. The critical question is: how do plants that are completely lacking this gene manage to retain their functional import machinery (de Vries et al., 2015)? Since Ycf1 is an essential protein in *Arabidopsis*, it is difficult to study protein import in knockout plants. Nonetheless, ecotypes of *Arabidopsis* can be grown on media containing spectinomycin, which is a specific inhibitor of plastid translation (Wirmer and Westhof, 2006). Under these conditions it could be shown that Ycf1 is truly absent in *Arabidopsis* plants, thus enabling to study its role in protein import (Bölter and Soll, 2016; Köhler et al., 2016). Presumably, the seed contains sufficient Ycf1 protein for the plants to germinate, and spectinomycin-induced signaling leads to compensatory mechanisms that ensure survival on the antibiotic. Interestingly, as these two studies show, precursor proteins that depend on the general protein import machinery are still efficiently imported into the plastids, thus excluding the role of Yfc1 as a constituent of the main protein channel. Furthermore, the nuclear-encoded Tic20 is also not detectable under spectinomycin treatment, implying a feedback mechanism between plastid and nucleus concerning the assembly of the 1MDa complex (Bölter and Soll, 2016). Instead of being a main translocation factor, Ycf1 could be involved in the assembly of a plastid fatty acid synthase (ACCase). Under spectinomycin, plants are also lacking the plastid-encoded subunit AccD but are able to complement for that loss by upregulating the expression and import of a nuclear-encoded and plastid-targeted protein (Acc2). This upregulation only appears if Ycf1 is strongly diminished, suggesting a functional role of Ycf1 in assembling the ACCase holoenzyme (Bölter and Soll, 2016). Recently, Ycf1 was shown to be a target of a nuclear-encoded translational activator named PBR1, which is important for thylakoid biogenesis, suggesting it could play a role in this process (Yang et al., 2016). Although a potential role of Ycf1 in protein import cannot entirely be excluded, more research is needed to clarify its functional role(s).

Besides the discrepancies concerning the main translocation machinery, additional TIC components have been identified which are called the redox regulon. This regulon includes the proteins Tic55, Tic62, and Tic32 (Stengel et al., 2009). Tic55 is a Rieske protein, while both Tic62 and Tic32 are dehydrogenases. All proteins have been found in complexes containing Tic110; specifically, Tic32 shows a direct interaction with the N-terminus of Tic110 (Hörmann et al., 2004; Stengel et al., 2009). The role of these redox regulon members will be discussed in detail below.

## Completion of the Translocation Process: The Stromal Chaperone System

Upon reaching the stroma, the preprotein translocation proceeds by removing the cTP and subsequently folding into an active structure. Four distinct destinations for the imported proteins are possible: stroma, IEM, thylakoids and thylakoid lumen. The mature protein is either re-inserted into the IEM or, due to a bipartite transit peptide, directed to the thylakoids using different sorting mechanisms for further processing and assembly (Schünemann, 2007). The removal of the cTP is carried out by a soluble SPP which is essential for plants (Richter and Lamppa, 1998; Trösch and Jarvis, 2011). Import is an energy-consuming process resulting from nucleotide-hydrolysis. Although the TOC members are able to hydrolyze GTP, this provides only the minimal energy required for the irreversible initiation of protein import and is not the driving force for sufficient and complete import, so the energy must originate from a different source. It has been shown that the energy is provided in the form of ATP, which is hydrolyzed by stromal chaperones, leading to a sufficient motor activity for preprotein crossing of the OEM and IEM of the chloroplast (Pain and Blobel, 1987). Various chaperones have been determined as being involved in the folding of proteins and/or consuming the required energy via ATP hydrolysis, mainly the chloroplast Hsp70, Hsp90, Hsp93 and Cpn60 (Kessler and Blobel, 1996; Akita et al., 1997; Nielsen et al., 1997; Inoue et al., 2013). However, Cpn60, the homolog of bacterial GroEL, is most likely exclusively involved in protein folding and assembly of the newly imported mature proteins, especially Rubisco (Goloubinoff et al., 1989).

Hsp93 (bacterial ClpC) is a member of the Hsp100 family, which itself belongs to the broader AAA+ family (ATPases associated with various cellular activities) (Moore and Keegstra, 1993). Hsp100 proteins contain one or two AAA+ domains, and are typically arranged into a hexameric structure with a central pore which is sufficient for protein threading (Rosano et al., 2011). *Arabidopsis* features two genes encoding for the isoforms Hsp93-V and Hsp93-III. Beside the putative function of providing energy coming from ATP hydrolysis, Hsp93 has been shown to be a regulatory chaperone for the Clp protease system, thus functioning in quality control and potential degradation of the incoming preproteins (Kovacheva et al., 2005).

Originally, three chloroplast Hsp70 isoforms in pea were reported. Two of them are located in the stroma whereas one is supposed to reside in the intermembrane space (Ratnayake et al., 2008). However, in *Arabidopsis* the gene coding for the latter has not yet been identified, leaving doubts about the existence or identity of such an intermembrane-space chaperone. *Arabidopsis* double null mutants of the stromal Hsp70 isoforms are embryo lethal and single mutants already exhibit biogenesis and import defects (Su and Li, 2010).

CpHsp90 was identified in complexes containing import intermediates at late import stages that also contain Tic110 and Hsp93 (Inoue et al., 2013). A specific and reversible Hsp90 ATPase inhibitor arrests protein import in chloroplasts, whereas initial binding to the TOC complex is not impaired, clearly emphasizing a role of cpHsp90 in late import stages (Nakamoto et al., 2014).

**FIGURE 3:**
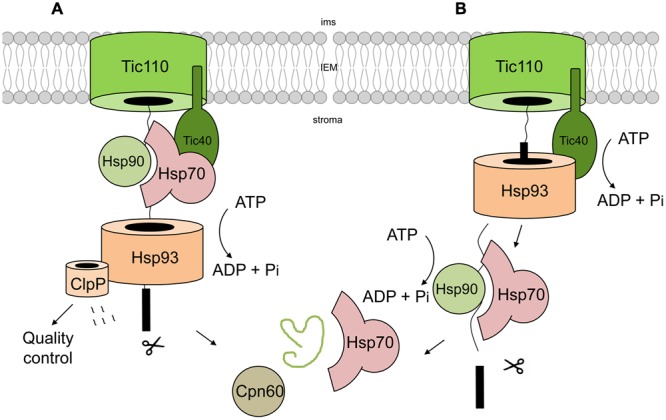
**The stromal chaperone system.** Two different models have been hypothesized concerning the main import motor of the chaperones. One model **(A)** involves a secondary function of Hsp93, assuming that this protein acts mainly in the quality control pathway by degrading mistargeted or wrongly folded proteins. In this model the main energy is consumed by Hsp70 and not by Hsp93 (Flores-Pérez et al., 2016). A recent study suggest that Hsp93 interacts subsequently with incoming preprotein at the N-terminal cTP, whereas Hsp70 binds to the mature parts of the protein (Huang et al., 2015). This enable the two chaperone systems to interact at least partially in parallel with the preproteins. After completing of the import by processing the cTP, proteins are folded with the help of various chaperones like Cpn60 and Hsp70 **(B)**.

Due to the complexity of the chaperone system in chloroplasts, there is an ongoing discussion about the specificity and import-related function of each individual chaperone, resulting in different models. It is still not completely clear which protein is the potential candidate to constitute the main motor protein for providing the import energy. In mitochondria and ER, the responsible driving force is believed to come from ATP hydrolysis performed by Hsp70 chaperones which are located in the matrix and lumen, respectively (Park and Rapoport, 2012; Dudek et al., 2013). Thus, it was long thought that cpHsp70s are likewise the main motor in chloroplasts. In that context, it seems logic that the responsible ATPase interacts directly with the incoming preproteins, or at least associates with the TIC translocon and for a long time, this scenario could not be shown for stromal Hsp70, hence it seemed unlikely that Hsp70 alone provides the required power. However, it could be shown in 2010 for the moss *P. patens* that Hsp70 is indeed involved in protein import into chloroplasts as a stromal Hsp70 co-immunoprecipitated with early-import intermediates, as well as with Tic40 and Hsp93 (Shi and Theg, 2010). In agreement with this, *Arabidopsis* mutants lacking the chloroplast isoforms of Hsp70 showed a reduced import level of preproteins, which could also be demonstrated in the moss *Physcomitrella patens* (Su and Li, 2010; Shi and Theg, 2010). Furthermore, it was suggested that the ATP requirements correlate with the activity of moss Hsp70, emphasizing the idea that cpHsp70 is the only energy-providing motor, at least in moss (Shi and Theg, 2010). Interestingly, *Arabidopsis* double mutants of Hsp93 and Hsp70 showed an additive effect in decreased import capacity compared to the single knockout mutants, leading to the theory that both proteins are acting at least partially in parallel as independent import players (Su and Li, 2010). This idea was somewhat supported later on: it was hypothesized that Hsp70 is the motor protein whereas Hsp93 is stably associated with the Clp protease complex at the IEM, suggesting a permanent role in quality control and degradation of preproteins and not a role in powering protein translocation (**Figure 3A**). In this study, the authors used a transgenic line in which the interaction of Hsp93 with the protease ClpP was disrupted, but the protein itself was still localized to the IEM and interaction with Tic110 was also ensured (Flores-Pérez et al., 2016). This enabled the study of the role of Hsp93 in protein import independent from its role in proteolysis. However, the truncated version could not complement the *hsp93* import defective phenotype, thus excluding the possibility of Hsp93 being the main motor functioning in protein import (Flores-Pérez et al., 2016).

In remarkable contrast to the above-mentioned observations, a recent study on that topic could show that Hsp93 directly binds to both the N-terminal cTP and the mature part of incoming preproteins, thus clearly indicating a role in early import stages and challenging the above-mentioned theory (Huang et al., 2015). These authors favor the hypothesis that both chaperones could prefer different regions of the preprotein and thus provide different modes of translocation force, which would result in additive import defects in the double mutants. This would also hold true if Hsp93 was to be the primary motor for the cTP and Hsp70 for the mature region (**Figure 3B**). Preprotein processing takes place during binding to Hsp93 and thus, binding to the mature protein is also detected. In their model, Hsp70 is entirely responsible for interacting with the mature protein, acting in parallel and one defined step after the action of Hsp93 (**Figure 3B**).

Taken together, and taking the described discrepancies into account, it remains unclear why the chloroplast evolved such a complex and divergent chaperone system in comparison to other subcellular compartments such as mitochondria or the ER. However, as the cytosolic chaperones display distinct preprotein affinities, it is still reasonable to say that the stromal counterparts do the same, while keeping the opportunity to react efficiently toward different import conditions resulting from potential environmental stimuli.

## Potential Involvement of Import Regulation in Plant Acclimation

Translocation efficiency of chloroplast proteins is highly dependent on post-translational modifications, enabling the plant to react quickly and efficiently toward external stimuli. The above-mentioned import steps can be influenced by various regulation mechanisms, including redox-mediated circuits of both cytosolic and stromal pathways, and phosphorylation-dependent activities.

### Redox-Sensing at the Outer Envelope Membrane

Redox-mediated communication and regulation within cellular processes had already been present in the prokaryotic ancestor, thus leading to a range of reduction- and oxidation-driven regulation in the organelle. One of the best-studied mechanisms in the bacterial ancestor is the bacterial disulfide bond (Dsb) that ensures accurate folding of periplastic proteins (Guddat et al., 1998). The central component is DsbA, which contains a highly redox-active CXXC motif and can bind to its substrate during protein import. Similar to this, a thiol-dependent oxidation mechanism has been addressed to the thylakoid lumen (Gopalan et al., 2004).

Since even mitochondrial intermembrane-destined proteins are imported in an oxidation-driven reaction by the mitochondrial disulfide relay (Herrmann et al., 2009), this might also be the case for chloroplast import. However, this field has only recently gained more attention. Redox-mediated regulation could be observed at different stages of the import process. Both in the OEM as well as in the IEM, import-involved proteins exhibit redox-active properties.

Import activity is highly stimulated *in vitro* upon the addition of reducing agents like DTT or TCEP, and impaired by oxidizing substrates, suggesting a potential role of cysteines and disulfide bridges (Stengel et al., 2009). Interestingly, all TOC components contain several conserved cysteines. Seven are found in Toc75, present in both vascular and non-vascular plants. The POTRA domain contains four of them. In the case of cytosolic-facing POTRA domains, these cysteines could be involved in redox-mediated regulation. Toc159 displays five cysteines; two of them are conserved and are located within the GTPase domain. One of these two is also present in the GTPase domain of Toc34 and is fairly exposed (Sun et al., 2002), suggesting a suitable target for redox reactions. In the last identified TOC component, Toc64, ten cysteines could be found, of which six are conserved through vascular and non-vascular plants.

**FIGURE 4:**
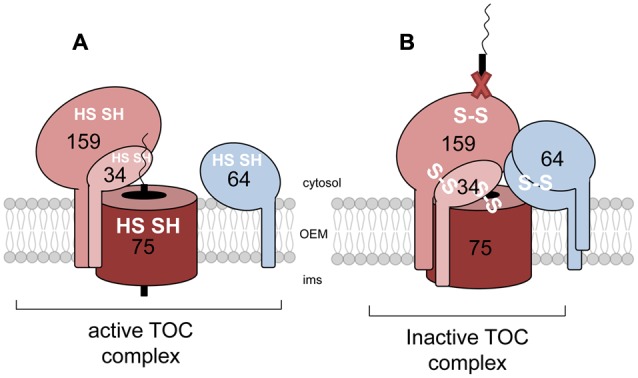
**Redox regulation at the outer envelope membrane.** Disulfide bridges between conserved cysteine residues of the TOC constituents are involved in the redox modulation of the constituents of the OEM. Under reducing conditions, the TOC receptors are loosely attached, thus forming the open and active TOC complex **(A)**. Upon oxidation due to various external stimuli the generated intra- and intermolecular disulfide bridges lead to a blocked TOC complex which inhibits import of precursor proteins either by blocking the channel or altering the binding capacity of the receptor toward the preproteins **(B)**.

In their reduced state, Toc159, Toc34 and Toc75 are loosely attached, harboring different reduced thiols and thus, forming the so-called ‘active’ TOC complex, prepared for preprotein recognition and binding (**Figure 4A**). Upon oxidation, intra- and intermolecular disulfide bridges are generated, commonly between Toc159, Toc34 and Toc75, resulting in a heteromeric TOC complex (Seedorf et al., 1995). Different hypotheses concerning the mode of action have been suggested. Oxidized on the one hand, this bulky complex could inhibit the import rate by simply blocking the channel and thus preventing the entrance of incoming proteins (**Figure 4B**). However, another mechanism suggests that not only does channel blocking occur, but also the preprotein-binding capacity of the receptor proteins is already altered as the cysteines are located within the preprotein binding, the GTPase, domain (Stengel et al., 2010). Up to now, all these experiments have been carried out by adding, reducing or oxidizing agents *in vitro*, and so far, a discrete physiological role is still missing. However, it is still reasonable to assume that changing environmental conditions led to different redox states in the cytosol, due to the production of reactive oxygen species for example, and hence affecting the redox modulation of the translocation apparatus.

### Redox Sensing at the Inner Envelope Membrane

Regarding the fact that the TOC complex could be regulated in a thiol-dependent mechanism, it can be supposed that this regulation is also effective for the translocase of the IEM.

**FIGURE 5:**
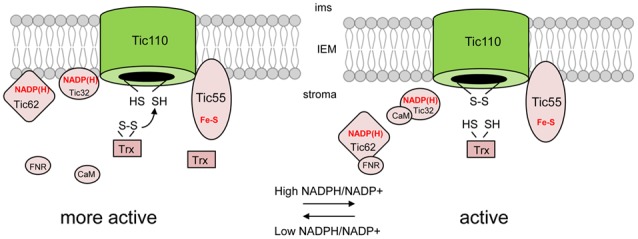
**Import regulation of the TIC complex from the stromal site.** Similar to the redox regulation at the OEM import of precursor proteins is accelatered under reducing conditions, suggestively due to an open conformation of the main channel, Tic110. A second regulation mechanism involves the stromal redox state, which is reflected by the NADPH/NADP^+^ ratio. A low NADPH/NADP^+^ ratio could be shown to enhance the import rate compared to a higher NADPH/NADP^+^ ratio.

Indeed, a thiol-dependent interaction between Tic110 and Tic40 has been observed, but its *in vivo* role has to be clarified (Stahl et al., 1999). Tic110 itself has been found to contain one or two regulatory disulfide bridges (Balsera et al., 2009). These intramolecular bridges could have a critical influence on the structure and function of the central TIC component. Switches between reduction and oxidation of these disulfide bridges could either lead to an open or closed formation of Tic110, respectively, and thereby limit the amount of incoming preproteins (**Figure 5**). The stromal thioredoxin family has been demonstrated to operate on disulfide bonds of Tic110 (Balsera et al., 2009). The redox state of thioredoxins is directly linked to both photosynthetic activity and other redox-dependent mechanisms in the organelle, thus it might act as a transport signal that eventually reaches the import machinery to regulate the chloroplast import rate. The intermembrane space protein Tic22 contains a conserved cysteine (Glaser et al., 2012), which could be involved in intramolecular disulfide bridges leading to dimerization of Tic22. Furthermore, since Tic110 exposes one cysteine into the IMS, a possible disulfide bond between the soluble Tic22 and the pore protein Tic110 during preprotein is also a hypothesis. However, no redox-mediated modulation has been reported so far and this hypothesis has to be addressed experimentally.

A direct read-out for the stromal redox state is the ratio between NADPH and NADP+. These reduction equivalents deliver electrons which are required for enzymatic activities of many biosynthetic pathways within the organelle. All the required enzymes for a subset of different pathways have to be imported at a specific rate depending on the actual need within the organelles. Therefore, protein import activity must be regulated according to these requirements, which could be mediated by the stromal redox state. Independent studies have shown that the stromal redox state influences the import activity of a subset of preproteins (Stengel et al., 2009; Zhang et al., 2016). Interestingly, several components, namely Tic62, Tic55 and Tic32, have been shown to associate dynamically with the core complex, leading to the assumption that these transient TIC components act in a regulatory mechanism in response to the stromal redox state (Stengel et al., 2008). One prominent candidate was Tic62, which showed a triple-localization pattern, shuttling from a membrane associated state at the IEM as well as the thylakoids to the stroma in response to changing NADP+/NADPH ratios, and thus being able to mediate signals from the photosynthetically active thylakoids to the import machinery (Stengel et al., 2008). Since one important function of Tic62 in vascular plants is the binding of the chloroplast-targeted ferredoxin-NADP(+) oxidoreductase (FNR) to these membranes via specific C-terminal motifs (Alte et al., 2010), its shuttling could significantly influence electron-transfer processes from this photosynthetic enzyme to different acceptor proteins which could display a signal transduction chain. As Tic62 possesses a NADPH binding site and acts as a dehydrogenase *in vitro*, it might also be involved in a direct electron transfer onto yet unknown acceptor proteins (Stengel et al., 2008). Furthermore, another binding partner of FNR, named Trol, has been characterized. This thylakoid-localized protein harbors a similar single C-terminal extension as is found repeatedly in Tic62 and was demonstrated to interact with FNR (Jurić et al., 2009; Lintala et al., 2014). It could be shown that Trol also associates with the IEM, thus it might also participate in the signal transduction chain involving Tic62/FNR (Jurić et al., 2009). Interestingly, the FNR binding C-terminal motif is exclusively found in vascular plants, leading to the assumption that this regulatory mechanism has evolved later in evolution. This might suggest that, for all other plants, the ecological pressure was not high enough to evolve a system that regulates their protein import activity in response to changing stromal redox conditions, which is in contrast to the old evolutionary regulation mechanism of thiol oxidation. A second protein possibly involved in redox regulation is Tic32, which is another member of a dehydrogenase family capable of transferring electrons. Like Tic62, the affinity toward the TIC complex is lower under reduced conditions. Interestingly, Tic32 is also subject to calmodulin/Ca2+ dependent regulation. It could be shown that calmodulin (CaM) directly binds to Tic32, which promotes import, and that specific inhibition of this interaction decreased import efficiency (Chigri et al., 2006). Thus, two very different modes of action can regulate the TIC translocon (**Figure 5**). The third member of the redox regulon is Tic55, a Rieske protein found in close proximity of Tic110. It is anchored to the IEM by two alpha helices and exposes its C-terminal region into the stroma. Recently, Tic55 was identified as a potential thioredoxin target by affinity chromatography on a Trx-column (Bartsch et al., 2008), which is supported by the presence of a CXXC motif. The molecular function of Tic55 is still unclear, but recently, a study was published in which a hydroxylation activity during leaf-senescence-dependent chlorophyll breakdown was demonstrated for Tic55 (Hauenstein et al., 2016). This potential function of Tic55 connects chlorophyll metabolism to the chloroplast import demand and could function as a coordinator of the chloroplast homeostasis, similar to GUN1, which is a mediator of retrograde signaling. Under stress conditions, when chlorophyll is degraded, Tic55 could relay the required information which would eventually reach the nucleus in order to respond efficiently toward external stimuli. All the presented import regulation mechanisms are clearly involved in fine-tuning of the process rather than representing a molecular on/off switch, since single knockout mutants of the redox regulon components have, so far, no reported defects in protein import (Bölter et al., 2015).

### Phosphorylation of the TOC Complex

The number of import sites per chloroplast was estimated, leading to different results: counting the number of radioactive mature proteins inside the organelle led to an estimated number of 3,500 (Friedman and Keegstra, 1989), whereas the approach using immunogold labeling of ultrathin sections with antibodies against main import components resulted in a higher number of 35,000 import sites (Morin and Soll, 1997). The discrepancy between these numbers can be explained by the fact that the immunogold labeling informs us about the total number of import complexes in the envelope, while the radioactive experiment gives us a measure of the fraction of these complexes that are actively importing. The switch between activity and non-activity of import complexes is likely to be modulated by the number of preproteins in need of being imported, amongst other signals.

It has been suggested that heterodimerization of the receptors, as well as their preprotein-binding capacity, is regulated by phosphorylation. PsToc34 and atToc33 are phosphorylated, whereas atToc34 is not, giving the opportunity to hypothesize that this represents specificity toward a different subset of preproteins (Jelic et al., 2003). The phosphorylation might negatively affect GTP and preprotein binding of the respective receptor, and the whole TOC integrity is negatively influenced by phosphorylation *in vitro* (Oreb et al., 2007).

Signals triggering phosphorylation are, however, still not well defined. Data from transgenic *Arabidopsis* mutant lines showed that a phosphomimicking mutant of atToc33 is indeed affected in import capacity, whereas a non-phosphorylatable version of atToc33 exhibited a WT-like phenotype (Aronsson et al., 2006; Oreb et al., 2007). The latter observation in particular clearly indicates that phosphorylation mediated regulation is not a common or permanent regulation mechanism during plant development but rather an on/off switch in response to either a short period of a developmental change or to different, yet undefined, external stimuli. This could be the case, for example, upon cold or high light stress where the protein demand in the chloroplast is changed, or a specific subset of proteins is required. Under these conditions, fast post-translational modification machinery is required and phosphorylation of the TOC receptors might represent a relevant and efficient target for such an event. Regulation could occur in two ways. On the one hand, the overall import rate is affected (reduced, if phosphorylated) by downregulating the affinity to preproteins. On the other hand, phosphorylation could change the TOC complex stability, which would lead to the import of a distinct subset of client proteins.

Under this aspect, it would make sense if the responsible kinase was located in close proximity to avoid long shuttling pathways and to ensure specificity. In pea chloroplasts a 98-kDa, ATP-dependent outer membrane-attached kinase was identified as the responsible kinase (Sveshnikova et al., 2000). However, an *Arabidopsis* homolog is still missing, thus the identity of the responsible kinase remains elusive. Besides atToc33 (psToc34), the Toc159 family is also a target for phosphorylation, which is putatively mediated by a 70-kDa, ATP-dependent kinase (Fulgosi and Soll, 2002). All members are highly phosphorylated in their variable A-domain, consequently leading to a distinct phosphorylation pattern, ranging from many phosphorylation sites (atToc159) to few (all others) (Agne et al., 2010). As the A-domain between the members already displays a heterogeneous profile in sequence characteristics, the phosphorylation event could either be irrelevant or, contrarily, even enhance specificity toward preproteins.

Besides having a direct effect, phosphorylation could also act as part of a signaling cascade or promote indirectly another post-translational mechanism, like ubiquitination. It has been shown that phosphorylation can indeed have a negative or positive effect on ubiquitination (Hunter, 2007). This would provide a link to a recently made observation. Ling and Jarvis (2016) identified an OEM E3 ubiquitin ligase (SP1), which upon abiotic stress marks TOC components for degradation. It must be clarified if phosphorylation enhances this effect, which would provide new insights into the regulation made by phosphorylation.

## Dedication

We dedicate this review article to the memory of Kentaro Inoue and his significant contributions to the chloroplast import field.

## Author Contributions

All authors listed have made substantial, direct and intellectual contribution to the work, and approved it for publication

## Conflict of Interest Statement

The authors declare that the research was conducted in the absence of any commercial or financial relationships that could be construed as a potential conflict of interest.
